# Editorial: Assessing the Safety of Thoracic Surgery Techniques for Non-small Cell Lung Cancer

**DOI:** 10.3389/fsurg.2022.859648

**Published:** 2022-03-07

**Authors:** Andrew E. Donaldson, Marco Scarci, Hasan Batirel, Christopher W. Seder

**Affiliations:** ^1^Department of Cardiovascular and Thoracic Surgery, Rush University Medical Center, Chicago, IL, United States; ^2^Department of Thoracic Surgery, San Gerardo Hospital, Monza, Italy; ^3^Department of Thoracic Surgery, Marmara University Hospital, Istanbul, Turkey

**Keywords:** lung cancer, complications, outcomes, segmentectomy, postoperative care

When reflecting on recent innovations in lung cancer treatment, many clinicians note medical therapies such as immune checkpoint inhibitors, which have demonstrated promising results. However, significant developments have been made in the surgical realm, including the advent of minimally invasive techniques, improvements in surgical technology, and enhancements in perioperative care. As advances in surgical therapy are investigated, it is critical to ensure that new therapies do not compromise patient safety or oncologic outcome. In this issue of *Frontiers*, a series of reports have been compiled focusing on the operative and perioperative treatment of non-small cell lung cancer (NSCLC).

Integral to the wide-spread adoption of minimally-invasive lung surgery was the development of the thoracoscopic stapler. Prior to this, fissures and lung parenchyma were commonly dissected using electrocautery. In this issue, Lu et al. report the results of a meta-analysis examining the safety of using a stapler versus electrocautery for pulmonary segmentectomy. While the cohort is small (*n* = 385; 6 studies), patients who underwent segmentectomy with electrocautery experienced more postoperative complications than those in which parenchymal stapling was used. Duration of surgery was equivalent between groups, and despite improvements in rate of air leak, pneumothorax, and chylothorax with stapling, chest tube drainage time was not different. It should be noted that each segmentectomy has its peculiarities and there are reports that re-expansion of the remaining lung may be influenced by the division technique (1). Thus, it is important to personalize technical preferences in case of atypical or large segmentectomies.

Since the mid-1990's, lobectomy has been considered the standard of care for resection of early-stage lung cancer (2). However, there has been a trend toward the selective application of sublobar resection due to mounting evidence suggesting equivalent oncologic outcomes and improved pulmonary reserve following surgery. What remains unclear is when should sublobar resections be used, what patients are most likely to benefit, and are all sublobar resections equivalent? These are interesting questions, especially considering that lung cancer incidence in patients over 65 years old is expected to increase (3). Zhang et al. attempt to shed some light on these questions by comparing survival between lobectomy, segmentectomy, and wedge resection in patients 75 years and older. Across all ages, lobectomy and segmentectomy demonstrated equivalent survival, while wedge resection had worse overall (OS) and lung cancer-specific survival (LCSS) compared to lobectomy. Interestingly, the authors found that the benefit of lobectomy over sublobar resection ceased to exist in patients older than 85 years. Until the results of the CALGB 140503 (4) and other ongoing randomized controlled trials examining sublobar resections mature, studies such as this provide some guidance for surgeons as they choose the best operation for each individual patient.

Also included, Zhou et al. report the feasibility and safety of atypical segmentectomy, an intermediate between conventional segmentectomy and wedge resection. The authors performed left anterior segmentectomy (S^3^+${\text{S}}_{\text{C}}^{1,2}$) *via* a two-port approach on 16 patients with cT_1_N_0_M_0_ tumors. They reported no perioperative morbidity or mortality and no recurrence in the 47.5-month average follow up period. This is an encouraging start to the investigation of a new surgical technique, but there are important considerations. In this study, it seems that performance of atypical segmentectomy with adequate margins depended on precise intraoperative tumor localization using a new technique: clock dial-integrated positioning (CDIP) (5). Although this method of localization was first described in 2015, it is not a standard modality of tumor localization that most surgeons have access to. Despite this, further investigation into this technique is warranted, as it may represent a promising advancement in lung cancer care.

In the setting of metastatic NSCLC, the role of surgery remains less defined. It has been well documented that oligometastatic disease may benefit from surgery if there is no mediastinal lymphatic involvement (6). Wang et al. investigate the role of lobectomy in the treatment of patients with distant metastatic disease. Among all stage IV patients, OS and LCSS were improved in those who underwent resection compared to those who did not. However, these findings could easily be confounded by individual tumor and metastasis characteristics. This draws attention to the fact that while surgery may benefit highly-selected patients with metastatic disease, those patients must be chosen very carefully.

In the final report in this series, Dong et al. examine the feasibility of discharging patients within 24 h of lung resection for early-stage NSCLC. The authors describe an extensive protocol to facilitate early discharge, including outpatient workup, preemptive family education, and daily phone calls for the first week postoperatively. They found that nearly all patients were discharged within 24 h, with no difference in postoperative complications and a low readmission rate. In an era increasingly focused on cost-savings, this is an important concept. However, it is possible that complication rates are underestimated in the early discharge group, as they do not receive close postoperative monitoring. The early discharge paradigm also relies heavily on the patient's home support structure, which may disqualify patients who live alone or have family who are less comfortable with providing care.

There is no doubt that advances in surgical technology, technique, and perioperative care have great potential to improve the outcomes of patients with lung cancer. If nothing else, the reports included in this issue of *Frontiers* reinforce that lung cancer care will continue to change and become increasingly individualized as time goes on.

## Author Contributions

All authors listed have made a substantial, direct, and intellectual contribution to the work and approved it for publication.

## Conflict of Interest

The authors declare that the research was conducted in the absence of any commercial or financial relationships that could be construed as a potential conflict of interest.

## Publisher's Note

All claims expressed in this article are solely those of the authors and do not necessarily represent those of their affiliated organizations, or those of the publisher, the editors and the reviewers. Any product that may be evaluated in this article, or claim that may be made by its manufacturer, is not guaranteed or endorsed by the publisher.
